# A dataset of Solicited Cough Sound for Tuberculosis Triage Testing

**DOI:** 10.1038/s41597-024-03972-z

**Published:** 2024-10-18

**Authors:** Sophie Huddart, Vijay Yadav, Solveig K. Sieberts, Larson Omberg, Mihaja Raberahona, Rivo Rakotoarivelo, Issa N. Lyimo, Omar Lweno, Devasahayam J. Christopher, Nguyen Viet Nhung, Grant Theron, William Worodria, Charles Y. Yu, Christine M. Bachman, Stephen Burkot, Puneet Dewan, Sourabh Kulhare, Peter M. Small, Adithya Cattamanchi, Devan Jaganath, Simon Grandjean Lapierre

**Affiliations:** 1 University of California San Francisco, School of Medicine, 533 Parnassus Ave, San Francisco, CA 94143 USA; 2 Sage Bionetworks, Seattle, WA 98103 USA; 3 CHU Joseph Rasera Befelatanana, Antananarivo, 101 Analamanga Madagascar; 4 Centre d’Infectiologie Charles Mérieux, Antananarivo, 101 Analamanga Madagascar; 5 CHU Tambohobe Fianarantsoa, Fianarantsoa, 301 Haute-Matsiatra Madagascar; 6 Université de Fianarantsoa, Fianarantsoa, 301 Haute-Matsiatra Madagascar; 7 Ifakara Health Institute, Environmental and Ecological Sciences & Interventions and Clinical Trials Departments, Kiko Avenue, Plot 463, Mikocheni, Dar es Salaam Tanzania; 8 Christian Medical College, Ida Scudder Road, Vellore, 632004 Tamil Nadu India; 9 National Tuberculosis Programme, 463 Hoang Hoa Tham, Ba Dinh District, Hanoi, Vietnam; 10 Stellenbosch University, Division of Molecular Biology and Human Genetics, Matieland, 7602 South Africa; 11 Walimu, Plot 5-7, Coral Crescent, Kololo, Kampala Uganda; 12 De La Salle Medical and Health Sciences Institute, Governor D. Mangubat Avenue, Dasmarinas Cavite, 4114 Philippines; 13 Global Health Labs, 14360 SE Eastgate Way, Bellevue, WA 98007 USA; 14 University of California Irvine, School of Medicine, 333 City Blvd. W Suite 400, Orange, CA 92868 USA; 15 Centre de Recherche du Centre Hospitalier de l’Université de Montréal, Immunopathology Axis, 900 St-Denis, Montréal, Québec, H2X 0A9 Canada; 16 Université de Montréal, Department of Microbiology, Infectious Diseases and Immunology, 2900 Edouard-Montpetit, Montréal, Québec, H3T 1J4 Canada; 17 Present Address: Koneksa Health, One World Trade Center 285 Fulton St. 77th Floor New York, New York, NY 10007 USA

**Keywords:** Tuberculosis, Data publication and archiving

## Abstract

Cough is a common and commonly ignored symptom of lung disease. Cough is often perceived as difficult to quantify, frequently self-limiting, and non-specific. However, cough has a central role in the clinical detection of many lung diseases including tuberculosis (TB), which remains the leading infectious disease killer worldwide. TB screening currently relies on self-reported cough which fails to meet the World Health Organization (WHO) accuracy targets for a TB triage test. Artificial intelligence (AI) models based on cough sound have been developed for several respiratory conditions, with limited work being done in TB. To support the development of an accurate, point-of-care cough-based triage tool for TB, we have compiled a large multi-country database of cough sounds from individuals being evaluated for TB. The dataset includes more than 700,000 cough sounds from 2,143 individuals with detailed demographic, clinical and microbiologic diagnostic information. We aim to empower researchers in the development of cough sound analysis models to improve TB diagnosis, where innovative approaches are critically needed to end this long-standing pandemic.

## Background & Summary

Tuberculosis remains the leading infectious disease killer globally, partly due to public health systems’ inability to accurately diagnose millions of infected individuals every year^1^. Insufficient access to high-quality TB screening and diagnosis is recognized as one of the most important gaps in the cascade of care^2^. Here we describe a cough sound database including detailed demographic, clinical and microbiologic information for the development of AI-based sound classification TB triage models. As the WHO’s End TB Strategy calls for intensified research and innovation including the discovery of new tools for community-based screening, digital cough monitoring and *Acoustic Epidemiology* could represent new tools that can help bend the TB pandemic curve and accelerate the achievement of global TB elimination goals^3–5^.

The “missing millions” of undiagnosed patients living with active TB disease represent an heterogeneous group including those who did not access triage or diagnosis testing or weren’t appropriately referred for effective treatment. Improving the accuracy, portability, point-of-care amenability and connectivity of diagnostic tools and algorithms would have significant value. Most health systems build their TB programs on a combination of complementary screening followed by diagnostic tests. The WHO’s target product profile (TPP) for a community-based TB triage test suggests that it should be at least 90% sensitive and 70% specific^6^. According to the 2021 WHO TB screening guidelines, symptom-based screening with questionnaires, including cough, is 42% sensitive^7^. Besides having poor accuracy these guidelines have operational challenges that impede its sustained and uniform implementation within resource-challenged TB programs. Other tools such as digital chest X-rays combined with computer-aided detection (CAD) algorithms have also been evaluated in the context of TB triage. This approach was shown to be highly sensitive but had variable specificity and remains difficult to deploy due to limited availability of chest X-ray platforms at primary-level health facilities^8^. Whether in the context of community-based outreach screening or healthcare facility-based evaluation prior to confirmatory testing, cough classification models could complement or replace other triage strategies including symptom-based screening.

We historically have been unable to objectively monitor cough sounds and consequently reduced this data-rich symptom into subjective and dichotomous information (e.g., cough versus no cough, chronic versus acute, better versus worse). Advances in acoustics and machine learning (ML) have enabled the identification and recording of human coughs in real-world acoustic environments (cough detection) as well as differentiation of coughs from patients with distinct clinical conditions or at different stages of disease (cough classification). As part of the emerging field of *Acoustic Epidemiology*, this has the potential to develop novel screening or diagnostic assays with simple digital recording devices, such as a smartphone, tablet or watch^5^. Proof-of-concept studies previously showed that cough associated with TB contains a specific acoustic signature which can be recognized by ML models. A study by *Pahar et al*. suggests that a cough-based TB screening model can discriminate TB cough sounds from those associated with other lung conditions with 93% sensitivity and 95% specificity, exceeding the WHO TPPs^9^. In a study combining cough sound analysis and patients’ clinical characteristics, *Yellapu et al*. report that ML can be used to detect TB with 90% sensitivity and 85% specificity^10^. A third study from *Sharma et al*. reported cough classification models with AUCs reaching 0.86^11^. Those pilot studies report on ML models which were designed on small datasets and were not validated in external populations. Given the potential impact on performance of local disease epidemiology and population ethnicity among other confounders, large and diverse cough datasets are needed to replicate those studies.

We collected and are here releasing a dataset including 733,756 cough sounds from 2,143 patients across 7 countries with accurately annotated demographic, clinical and microbiologic diagnostic information. These data were initially used to enable and evaluate the CODA TB DREAM Challenge which invited participants to develop algorithms for prediction of TB diagnosis. The training data are now available for general use, and researchers are invited to leverage acoustic and clinical data to further develop and evaluate sound classification models for TB screening against a held-out test partition^12^. We aim to enable the development of models which could achieve the WHO TPP performance targets for the current ‘community-based TB triage test’ or the forthcoming TPP for a TB screening test^6,13^. This data set has limitations which include some selection bias since it was collected from a symptomatic presumptive TB population. The models which will be developed may hence not perform as well if used for asymptomatic screening at population level. Accordingly, more data should be collected from community screening activities.

## Methods

### Participants

A total of 2,143 participants were recruited from two parent studies described below. To be eligible, participants had to be 18 years or older and have a new or worsening cough for at least two weeks. took place at outpatient clinics in India, Madagascar, the Philippines, South Africa, Tanzania, Uganda, and Vietnam. All participants provided informed consent. A summary of participant demographics and country distribution are available in Supplementary table 1.

#### Rapid Research in Diagnostic Development TB network (R2D2 TB Network) study

The R2D2 TB Network study evaluates novel TB diagnostics in various stages of development among people with presumptive TB in five low- and middle-income countries: Uganda, South Africa, Vietnam, the Philippines and India^14^. Ethical approval for this study was obtained from institutional review boards (IRB) in the US and in each study site. In the US, approval was obtained from the University of California San Francisco IRB (# 20-32670). In Vietnam, approval was obtained from the Ministry of Health Ethical Committee for National Biological Medical Research (94/CN-HĐĐĐ), the National Lung Hospital Ethical Committee for Biological Medical Research (566/2020/NCKH) and the Hanoi Department of Health, Hanoi Lung Hospital Science and Technology Initiative Committee (22/BVPHN). In India, approval was obtained from Christian Medical College IRB (13256). In South Africa, approval was obtained from Stellenbosch University Health Research Ethics Committee (17047). In Uganda, approval was obtained from Makerere University, College of Health Sciences, School of Medicine, Research Ethics Committee (2020-182). In the Philippines, approval was obtained from De La Salle Health Sciences Institute Independent Ethics Committee (2020-33-02-A).

#### The Digital Cough Monitoring for screening, diagnosis and clinical follow-up of tuberculosis and other respiratory diseases project

This project was designed to embed digital cough monitoring within existing health facility-based TB diagnostic cohorts in Madagascar and Tanzania. Ethical approval for this study was obtained from institutional review boards (IRB) in Canada and in each study site. In Canada, approval was obtained from the Centre de Recherche du Centre Hospitalier de l’Université de Montréal IRB (# 2021-9270, 20.226). In Madagascar, approval was obtained from the Comité d’Éthique à la Recherche Biomédicale (IORG0000851 - N°051-MSANP/SG/AMM/CERBM).). In Tanzania, approval was obtained from the Ifakarah Health Institute IRB (31-2021) and the National Institute for Medical Research (NIMR/HQ/R.8a/Vol IX/3805).

### Data collection

#### Demographic and clinical data

At enrollment into the parent studies, participants underwent a baseline questionnaire, clinical examination, and sputum collection for TB testing. Study staff also recorded participants’ age, gender, height, weight, smoking status and duration of cough. HIV diagnosis was made either based on participant self-report of a positive HIV diagnosis or a positive test result. A summary of the available variables is shown in Supplementary table 2.

#### TB reference standard testing

Both Xpert MTB/RIF Ultra PCR and mycobacterial culture (Lowenstein-Jensen solid medium or MGIT liquid medium) were performed on sputum collected from all participants. Any participant whose first sputum Xpert MTB/RIF Ultra result was indeterminate or trace-positive, received a second sputum Xpert MTB/RIF Ultra test. Results from those assays were combined to determine TB status according to two reference standards: a microbiologic reference standard and a sputum Xpert reference standard. The sputum Xpert reference standard is restricted to Xpert MTB/RIF Ultra results on sputum samples. The microbiologic reference standard includes culture results, allowing for more individuals to be classified as TB positive. The microbiologic reference standard is considered the primary reference standard. Full details of the reference standards are described in Table 1.

**Table 1:** Reference standard definitions.

Result	Microbiologic Reference Standard	Sputum Xpert Reference Standard
TB positive	One or more of the following: • a positive sputum Xpert result • a positive urine Xpert result • a positive liquid culture result • a positive solid culture result • a positive Xpert Ultra results on contaminated liquid culture • two trace positive Xpert Ultra results on any sample type.	One of the following: • a positive sputum Xpert result • two trace-positive sputum Xpert Ultra results
TB negative	No positive results on any Xpert or Xpert Ultra testing and one of the following conditions: • two negative liquid cultures • two negative solid cultures • one negative liquid and one negative solid cultures	A negative sputum Xpert result
Indeterminate	No positive results on any Xpert or Xpert Ultra testing and only one trace result on Xpert Ultra testing of any sample type	An indeterminate or trace-positive sputum Xpert result followed by a non-positive result on repeat sputum Xpert Ultra testing

#### Cough recording

Cough sounds were collected using smartphones loaded with the Hyfe research app^15^. Specific phone models used in the different participating sites are presented in Supplementary Materials 1. Hyfe research app is designed to listen for explosive sounds corresponding to putative cough sounds. Once such an event has been found, a 0.5 second audio segment in selected, starting at the onset. If that segment includes a cough sound and that cough lasts for more than 0.5 second, only the first 0.5 second part of it is held. If more than one explosive phase are recognized in that segment (for example, two short, consecutive coughs), then the app will create two 0.5 second segments, one starting at the onset of the first explosive phase (and possibly including part of the second explosive phase), and another 0.5 second segment starting at the onset of the second explosive phase. The app uses a sampling rate of 44.1 kHz and a bit depth is 32 bits, floating point precision. Hyfe research app uses a server-based convolutional neural network (CNN) model to classify explosive sounds as coughs and recordings of these cough sounds are saved on a protected health information (PHI)-regulated server for analysis. Smartphones were positioned on tripods in rooms within the clinic in a dedicated room without background noise. Participants were asked to cough five times (solicited cough) while standing 60–90 cm from the tripod; participants who managed to produce at least three coughs were retained in the dataset. Some participants produced more than five coughs due to a triggered coughing fit and those additional coughs were also collected and included in the dataset. Solicited and triggered coughs could not be labeled distinctively and are treated the same in the dataset. For participants diagnosed with TB, all solicited coughs were collected prior to treatment initiation. After enrollment and onboarding, a subset of participants (n = 565) were also asked to carry a study phone for two weeks and collect longitudinal coughs sounds in an outpatient setting. Those sounds are labeled as longitudinal and made available within the dataset. Contrarily to the solicited cough recording component of this study, longitudinal cough recording was unsupervised by study personnel and was performed in community setting. For TB positive participants, longitudinal cough recording and TB treatment were both initiated as soon as possible following TB diagnosis but in no specific order. Patients could have been initiated on therapy prior to the start of recording. Contrarily to solicited coughs, longitudinal coughs sounds were recorded in an unsupervised approach in community settings where the Hyfe research app performance for cough detection has not been validated. The quality of the longitudinal cough data could be limited due to factors such as variation in the positioning of the recording device in relation to participants, background noise or presence of coughing individuals in the proximity of research participants. A tally of solicited and longitudinal cough sounds per data partition are available in Table 2.

**Table 2:** Number of cough recordings per data partition.

	Training set	Testing set
Solicited coughs (Participants)	9,772 (1,105)	9,062 (1,038)
Longitudinal coughs (participants)	714,922 (565)	0 (0)

Longitudinal coughs are not used for evaluation on the testing set, so are not quantified here.

### Data partitioning

The dataset was split into a training (n = 1,105) and validation set (n = 1,038). The dataset was randomly partitioned evenly between the training and testing set at the level of the participant (i.e., all of a participant’s cough sounds are in either the training or validation set). Since longitudinal coughs were collected in an unsupervised setting with the previously described limitations, longitudinal cough sounds were not included in the validation dataset.

### Data Pre-Processing

#### Cough sounds

The sound recordings available in this dataset have not undergone pre-processing beyond their identification as a cough sound by the Hyfe research app CNN model.

#### Clinical data

Data from all participating sites were collected with standardized questionnaires and definitions. Data formatting was harmonized in the open access database.

### Dataset description

Sage Bionetworks independently verified the variable balance between the training and validation sets as demonstrated in Supplementary table 1. A breakdown of key demographics and microbiologic reference standard results by country are shown in Table 3.

**Table 3:** Key variables summarized by country.

	India n = 241	Madagascar n = 234	Philippines n = 388	South Africa n = 275	Tanzania n = 197	Uganda n = 487	Vietnam n = 321
**Sex**							
Female	100 (41%)	115 (49%)	208 (54%)	144 (52%)	86 (44%)	203 (42%)	121 (38%)
Male	141 (59%)	119 (51%)	180 (46%)	131 (48%)	111 (56%)	284 (58%)	200 (62%)
**Age**							
Median [Q1, Q3]	47 [34, 58]	32 [24, 51]	39 [26, 53]	40 [32, 49]	40 [31, 50]	32 [26, 42]	54 [41, 64]
**Duration of cough (days)**							
Median [Q1, Q3]	45 [30, 90]	30 [17, 60]	21 [15, 30]	21 [14, 28]	30 [14, 30]	30 [16, 60]	30 [30, 90]
**Prior TB**							
No	204 (85%)	207 (88%)	325 (84%)	170 (62%)	147 (75%)	427 (88%)	258 (80%)
Not sure	0 (0%)	0 (0%)	4 (1.0%)	0 (0%)	0 (0%)	0 (0%)	1 (0.3%)
Yes	37 (15%)	27 (12%)	59 (15%)	105 (38%)	50 (25%)	60 (12%)	62 (19%)
**Microbiologic reference standard**							
TB Negative	216 (90%)	131 (56%)	348 (90%)	223 (81%)	161 (82%)	313 (64%)	199 (62%)
TB Positive	25 (10%)	103 (44%)	40 (10%)	52 (19%)	36 (18%)	174 (36%)	122 (38%)

## Data Records

De-identified participant demographic and clinical data, including TB reference standard results, cough sound WAV files, and a datafile linking participant IDs to sound file IDs were exported to a dedicated project in Synapse. Synapse is a general-purpose data and analysis sharing service where members can work collaboratively, analyze data, share insights, and have attributions and provenance of those insights to share with others. Synapse is developed and operated by Sage Bionetworks^16^. A total of 1,105 participants’ data are made available for access and download as a training dataset. The validation set is withheld, but models can be evaluated against the validation set via the instructions provided in the Synapse project.

All training set files are stored and are accessible via the Synapse platform with associated metadata and documentation and can be accessed at the following URL: 10.7303/syn31472953^12^.

## Technical Validation

All cough collection periods were observed by study staff and cough sounds were spot-checked for accurate recording. Patient metadata was reviewed by study staff for accuracy. The data described in this article were collected using the Hyfe Research app which uses a proprietary algorithm to identify cough sounds^15^. This model was previously shown to be 96% sensitive and 96% specific for cough detection using human-labeled sounds as a reference standard^17^. Using this algorithm, the probability of explosive sounds being human coughs is calculated and reported as a 0 to 1 probability score. We used a prediction score of 0.8 from this algorithm to filter potential non-cough sounds. To validate the precision of the Hyfe algorithm, a standalone computer vision and deep learning model was trained using Log Mel spectrogram images from ESC-50 and Coswara datasets^18,19^. The “VGG16” CNN based pre-trained model was trained for accurate classification of cough sounds and achieved a model accuracy of approximately 96% on Hyfe cough recordings^20^. Most of the recordings that were classified incorrectly had a Hyfe prediction score less than 0.8.

## Usage Notes

Users can register to evaluate predictive models of TB diagnosis against the held-out test partition via the instructions on the Synapse project. The scoring mechanism can evaluate two different types of models: (1) those that use only cough sounds, or (2) those which also incorporate clinical metadata variables which have been provided in the training dataset (sex, age, height, weight, reported duration of cough, prior TB diagnosis and type, hemoptysis, heart rate, temperature, weight loss, smoking in the last week, fever and night sweats). Models are submitted to the scoring queues as Docker images. Full instructions and example code is available on Synapse project website 10.7303/syn31472953^12^.

### Downloading the data

Given the number of files represented in the data, users should consider downloading the data via one of the programmatic Synapse clients (available in R or Python). For convenience, Python code for downloading the data is provided in the Synapse project wiki. The training dataset size is (0.43 GB) for the solicited coughs and (31.6 GB) for the longitudinal coughs. All training set files are stored and are accessible via the Synapse platform with associated metadata and documentation and can be accessed at the following URL: 10.7303/syn31472953.

### Data use agreement

To access the data, individuals must become Certified and Validated users of Synapse and maintain an active account on Synapse: http://www.synapse.org. They must also submit an Intended Data Use Statement and agree to the Terms of Use of the dataset. Terms of Use are summarized in Supplementary Materials 2.

## Supplementary information

CODATB_Supplementary_datadescriptor_V1_2024.07.22

## Data Availability

No additional data processing was conducted other than what has been described above.
