# Supplementary material for: A dataset of Solicited Cough Sound for Tuberculosis Triage Testing
Source: Sci Data. 2024 Oct 18;11:1149. doi: 10.1038/s41597-024-03972-z (PMC11489852; doi:10.1038/s41597-024-03972-z)
Supplement: Supplementary file 1 — CODATB_Supplementary_datadescriptor_V1_2024.07.22 [file 41597_2024_3972_MOESM1_ESM.docx]

**SUPPLEMENTARY MATERIALS**

## Supplementary materials 1 - Phone models used in the different participating sites

India

- Redmi 9 Prime
- Realme Narzo20

Madagascar

- Motorola G9 play

Philippines

- Myphone myWX2 Pro
- Xiaomi 9C

South Africa

- Nokia 3.1
- Nokia 5.4
- Xiaomi Redmi 9A

Tanzania

- Nokia 3.4 Ta-1288

Uganda

- Motorola G16
- Samsung M11
- Nokia model 5.3

Vietnam

- OPPOA54

## Supplementary materials 2 – Data Use Agreement

Researchers wishing to access the data must:

- You must reaffirm your commitment to the Synapse Pledge and must abide by the guiding principles for responsible research use and data handling within the Synapse Commons Platform as described in the Synapse Governance documents.
- You will not attempt to establish the identity of, or attempt to contact any of the subjects included in the data.
- You confirm that if you inadvertently receive identifiable information or otherwise identify a subject, you will promptly notify the ACT by emailing [act@sagebase.org](mailto:act@sagebase.org).
- You agree to establish appropriate administrative, technical, and physical safeguards to prevent unauthorized use of or access to the Data.
- You will report any data misuse or breach of data security to ACT by emailing [act@sagebase.org](mailto:act@sagebase.org).
- You will use the data only as identified in your intended data use statement (IDU), submitted through Synapse. The IDU should be written in English and must describe the objectives of the proposed research and study design and analysis plan (500 word maximum).
- Data accessors must acknowledge the following in all publications or presentations as follows:
  “The datasets used for the analyses described were contributed by Dr. Adithya Cattamanchi at UCSF and Dr. Simon Grandjean Lapierre at University of Montreal and were generated in collaboration with researchers at Stellenbosch University (PI Grant Theron), Walimu (PIs William Worodria and Alfred Andama); De La Salle Medical and Health Sciences Institute (PI Charles Yu), Vietnam National Tuberculosis Program (PI Nguyen Viet Nhung), Christian Medical College (PI DJ Christopher), Centre Infectiologie Charles Mérieux Madagascar (PIs Mihaja Raberahona & Rivonirina Rakotoarivelo), and Ifakara Health Institute (PIs Issa Lyimo & Omar Lweno) with funding from the U.S. National Institutes of Health (U01 AI152087), The Patrick J. McGovern Foundation and Global Health Labs.”

**SUPPLEMENTARY TABLES**

## Supplementary table 1 – Participant demographics across training and test sets

|  | **Training set (N=1105)** | **Testing set (N=1038)** | **Complete set (N=2143)** |
| --- | --- | --- | --- |
| **Sex** |  |  |  |
| Female | 517 (46.8%) | 460 (44.3%) | 977 (45.6%) |
| Male | 588 (53.2%) | 578 (55.7%) | 1166 (54.4%) |
| **Age** |  |  |  |
| Median [Q1, Q3] | 40.0 [28, 53] | 40.0 [29, 53] | 40.0 [28, 53] |
| **Height (cm)** |  |  |  |
| Median [Q1, Q3] | 162 [155, 168] | 162 [156, 168] | 162 [156, 168] |
| **Weight (Kg)** |  |  |  |
| Median [Q1, Q3] | 55.0 [49, 65] | 56.0 [49, 65] | 55.8 [49, 65] |
| **HIV status** |  |  |  |
| Negative | 878 (79.5%) | 809 (77.9%) | 1687 (78.7%) |
| Positive | 162 (14.7%) | 155 (14.9%) | 317 (14.8%) |
| Unknown | 65 (5.9%) | 74 (7.1%) | 139 (6.5%) |
| **Duration of cough (days)** |  |  |  |
| Median [Q1, Q3] | 30.0 [16, 60] | 30.0 [16, 50] | 30.0 [16, 60] |
| **Prior TB** |  |  |  |
| No | 903 (81.7%) | 835 (80.4%) | 1738 (81.1%) |
| Not sure | 3 (0.3%) | 2 (0.2%) | 5 (0.2%) |
| Yes | 199 (18.0%) | 201 (19.4%) | 400 (18.7%) |
| **Country** |  |  |  |
| India | 119 (10.8%) | 122 (11.8%) | 241 (11.2%) |
| Madagascar | 159 (14.4%) | 75 (7.2%) | 234 (10.9%) |
| The Philippines | 198 (17.9%) | 190 (18.3%) | 388 (18.1%) |
| South Africa | 137 (12.4%) | 138 (13.3%) | 275 (12.8%) |
| Tanzania | 87 (7.9%) | 110 (10.6%) | 197 (9.2%) |
| Uganda | 242 (21.9%) | 245 (23.6%) | 487 (22.7%) |
| Vietnam | 163 (14.8%) | 158 (15.2%) | 321 (15.0%) |
| **Microbiologic reference standard** |  |  |  |
| TB Negative | 807 (73.0%) | 782 (75.3%) | 1589 (74.1%) |
| TB Positive | 297 (26.9%) | 256 (24.7%) | 553 (25.8%) |
| **Sputum Xpert reference standard** |  |  |  |
| Indeterminate | 4 (0.4%) | 2 (0.2%) | 6 (0.3%) |
| TB Negative | 839 (75.9%) | 808 (77.8%) | 1647 (76.9%) |
| TB Positive | 262 (23.7%) | 226 (21.8%) | 488 (22.8%) |

## Supplementary table 2 - Available demographic, clinical and microbiologic variables

| **Variable** | **Values** | **Format/Definition** |
| --- | --- | --- |
| Country | Philippines Vietnam South Africa Uganda India Madagascar Tanzania | Country where the participant was enrolled |
| Sex | Male Female | Sex at birth reported by participant |
| Age | Numeric | Age calculated as date of collection - date of birth if known. If date of birth is unknown, reported age at time of collection. |
| Height | Numeric | Height in centimeters |
| Weight | Numeric | Weight in Kg |
| HIV status | Positive Negative | HIV status. All participants who do not report being HIV-positive receive HIV testing (using capillary or venous blood) Positive = Positive reported by patient or positive on a HIV test  Negative = Negative on a HIV test |
| Reported duration of cough | Numeric | Self-reported duration of current cough (days). At baseline, we ask: How many days have you had this new cough or cough that has been worse? |
| Prior TB | Yes No | Self-reported. At baseline, we ask: Have you ever had or been told you had tuberculosis (TB)? |
| Prior TB type: Pulmonary | Checked Unchecked | Asked at baseline: "With what kind of TB were you diagnosed?" (may select more than one) Participant selected pulmonary TB. |
| Prior TB type: Extrapulmonary | Checked Unchecked | Asked at baseline: "With what kind of TB were you diagnosed?" (may select more than one) Participant selected extrapulmonary TB. |
| Prior TB type: Unknown | Checked Unchecked | Asked at baseline: "With what kind of TB were you diagnosed?" (may select more than one) Participant selected Unknown. |
| Hemoptysis | Yes No | In the past 30 days, have you ever coughed up blood? |
| Heart rate | Numeric | Participant's heartrate (beats per minute) measured at baseline |
| Temperature | Numeric | Participant's temperature (Celsius) measured at baseline. |
| Weight loss | Yes No | Subjective, Self-reported. At baseline, we ask: In the past 30 days, have you experienced any weight loss? |
| Smoked in last week | Yes No | Asked at baseline "Have you used combustible tobacco and/or vaping products in the last 7 days?" |
| Fever | Yes No | Self-reported fever at baseline: "In the past 30 days, have you ever felt or experienced fever?" |
| Night sweats | Yes No | Self-reported night sweats at baseline: "In the past 30 days, have you ever experienced night sweats?" |
| Microbiologic reference standard* | TB Positive TB Negative Indeterminate | TB diagnosis based on sputum and culture results |
| Sputum Xpert reference standard* | Positive Negative Indeterminate | TB diagnosis based on sputum results alone |
| Xpert combined semi-quant | Trace Very Low Low Medium High | Highest semiquantitative result from sputum Xpert Ultra test conducted at baseline visit.  A semiquantitative result will only be available if the test was positive. |
